# Supplementary material for: Polymorphisms in Pfkelch13 domains before and after the introduction of artemisinin-based combination therapy in Southwest Nigeria
Source: PLoS One. 2025 Mar 31;20(3):e0316479. doi: 10.1371/journal.pone.0316479 (PMC11957316; doi:10.1371/journal.pone.0316479)

Samples: 13495
Bases: 822
Average spacing: 17

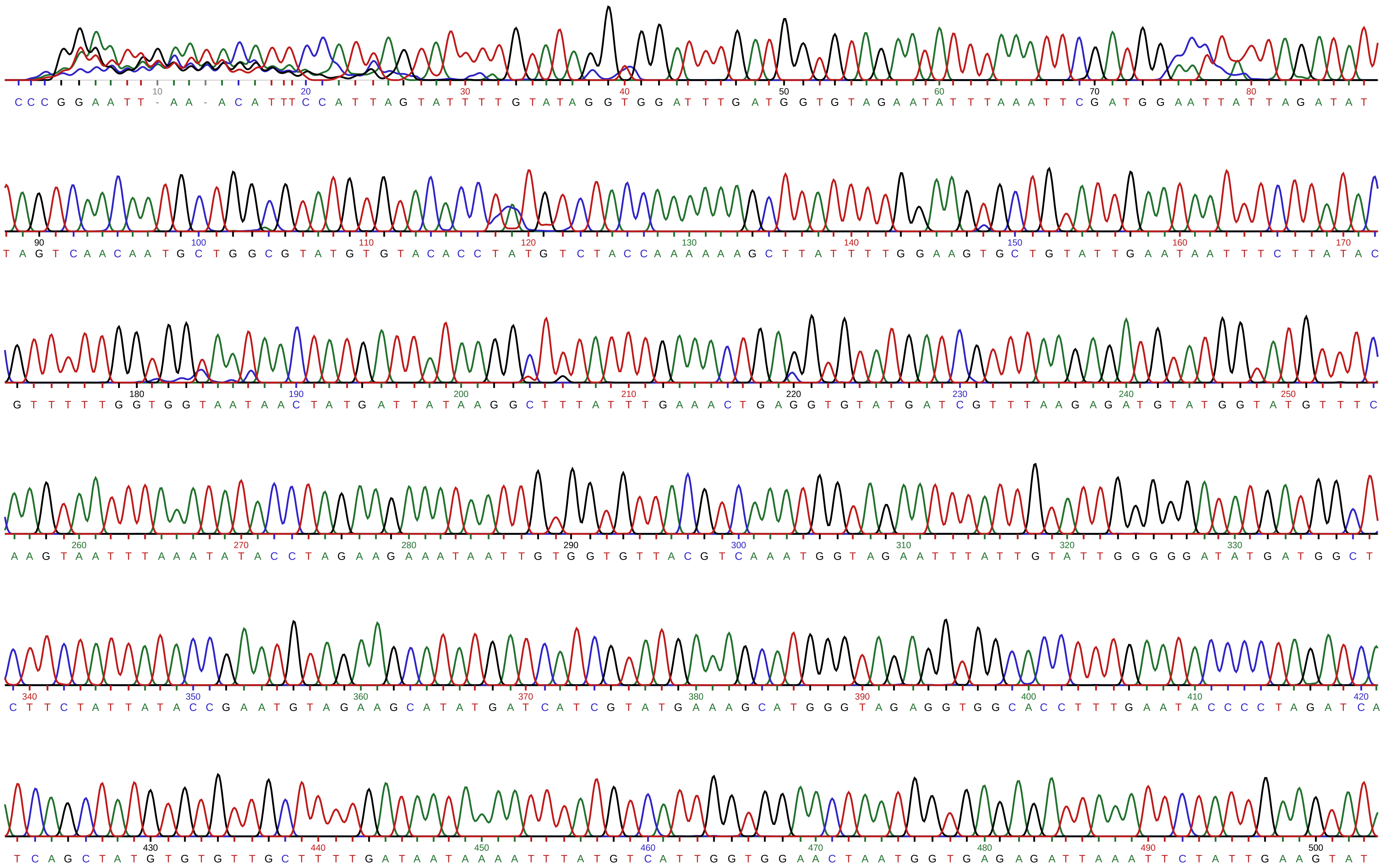

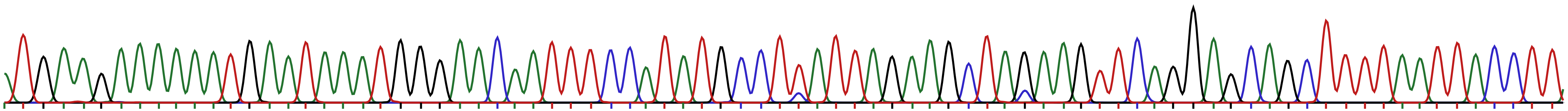

510 520 530 540 550 560 570 580
A T G A A G A A A A A A T G A A T A A A T G G G A A C A A T T T C C A T A T G C C T T A T T A G A A G C T A G A A G T T C A G G A G C A G C T T T T A A T T A C C T T

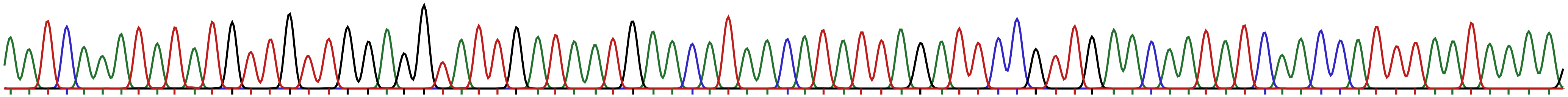

590 600 610 620 630 640 650 660
A A T C A A A T A T A T G T T G T T G G A G G T A T T G A T A A T G A A C A T A A C A T A T T A G A T T C C G T T G A A C A A T A T C A A C C A T T T A A T A A A A

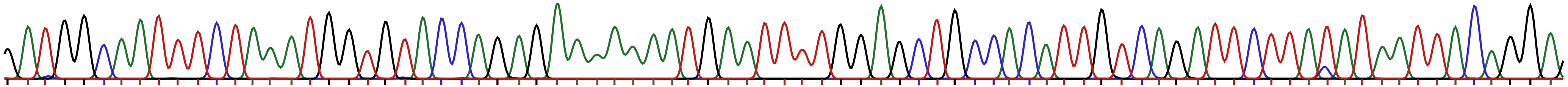

670 680 690 700 710 720 730 740 750
G A T G G C A A T T T C T A A A T G G T G T A C C A G A G A A A A A A A T G A A T T T T G G A G C T G C C A C A T T G T C A G A T T C T T A T A T A A T T A C A G G A

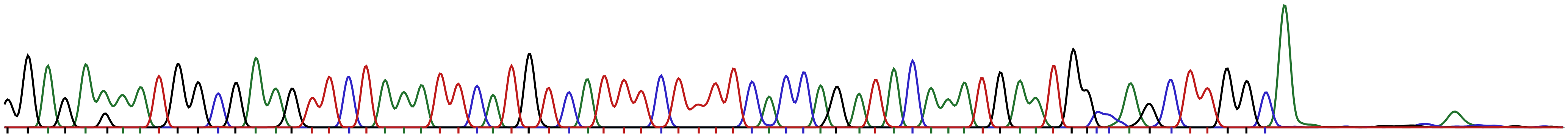

760 770 780 790 800 810 820
G G A G A G A A T G G C G A A G T T C T A A A T T C A T G T C A T T T C T T T T C A C C A G A T A C A A A T G A A T G G C C A G C T T G G C

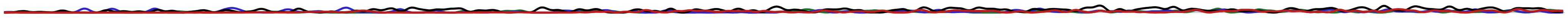

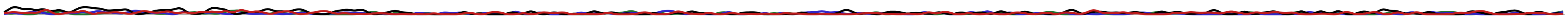

Samples: 13495
Bases: 822
Average spacing: 17

8/17/2022

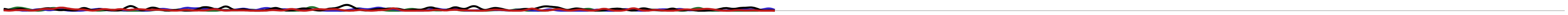

Supplement: Supporting information 2 — (ZIP) [file pone.0316479.s002.zip › 142KN1F_PREMIX_Plate_KELCH1_A01.pdf]
